# Instantaneous “catch‐and‐kill” inactivation of SARS‐CoV‐2 by nitride ceramics

**DOI:** 10.1002/ctm2.212

**Published:** 2020-10-18

**Authors:** Giuseppe Pezzotti, Eriko Ohgitani, Masaharu Shin‐Ya, Tetsuya Adachi, Elia Marin, Francesco Boschetto, Wenliang Zhu, Osam Mazda

**Affiliations:** ^1^ Ceramic Physics Laboratory Kyoto Institute of Technology Kyoto Japan; ^2^ Department of Immunology Graduate School of Medical Science Kyoto Prefectural University of Medicine Kyoto Japan; ^3^ Department of Dental Medicine Graduate School of Medical Science Kyoto Prefectural University of Medicine Kyoto Japan

Dear Editor,

We propose a nontoxic, sustainable alternative to conventional surface disinfection, possibly useful in fighting the present COVID‐19 pandemics. The global spread of COVID‐19 has increased awareness of how the SARS‐CoV‐2 virus is transmitted on surfaces.^1^ Person to person contagion can occur through contact with contaminated surfaces. To limit this contagion pathway, regular surface disinfection is recommended. Research indicates that this virus can remain viable for 4 to 72 hours on plastic, copper, and steel, and up to 7 days on surgical mask material,^2^ creating increased transmission risk in social and medical environments. Presently, the application of ethanol in combination with sodium hypochlorite or hydrogen peroxide or the use of ultraviolet surface irradiation effectively inactivates the virus. However, the practical application of these methods, as well as other antiviral protocols, is hindered by their toxic impact on human health.^3^ It is vital to develop surfaces, fabrics, and other materials that could inherently inhibit viral spread while concurrently being safe for humans.

One such material is silicon nitride (Si_3_N_4_), an FDA‐cleared bioceramic, which may be used in the human body. It has superior antibacterial behavior and has been proven safe for long‐term use in humans. It possesses a unique surface biochemistry that inhibits bacterial infections by long‐term elution of nitrogen (promptly converted into ammonia) in minute concentrations that, unlike bacteria and viruses, mammalian cells can easily metabolize.^4^ Within 1 minute, influenza A and enterovirus were completely inactivated by Si_3_N_4_ bioceramic particles suspended in water.^5^

In this study, we exposed SARS‐CoV‐2 virions to the above bioceramic as well as to aluminum nitride (AlN) micrometric powders suspended in water. The nitrogen‐based ceramic, AlN, undergoes surface hydrolysis analogous to that of Si_3_N_4_ when in such a solution. We used two controls, namely, a copper (Cu) particle suspension (a positive control, known to strongly inactivate pathogens and viruses^6^) and a negative control expected to have no effect, H_2_O. The supernatant virions were then inoculated into VeroE6/TMPRSS2 cells. We expected comparable antiviral behavior for Si_3_N_4_ and AlN, as these nitride compounds share the chemical similarity of N atoms with strong electronegativity.

Figure 1A shows results for TCID_50_ assay in case of virions exposed to Si_3_N_4_, AlN, and Cu powders in 15 wt.% at 1‐minute inactivation time. Compared with the water‐exposed negative control (sham sample), these three powders produced equally effective inactivation of SARS‐CoV‐2 virions (>99%). We then examined fragmentation of viral RNA upon 1‐minute contact with the powders by means of RT‐PCR experiments on the virions N‐gene sequence (Figure 1B). Unlike the case of powder‐unexposed control supernatant (sham sample), the viral RNA underwent nearly complete fragmentation when exposed to Cu, and was significantly damaged after both AlN and Si_3_N_4_ contact. Viral RNA on pelleted powders, after 1‐minute exposure, was not detectable for any of the three powders (Figure 1B). Experiments repeated at 10‐minute exposure revealed substantial RNA cleavage for all powders tested (see Supporting Information).

**FIGURE 1:**
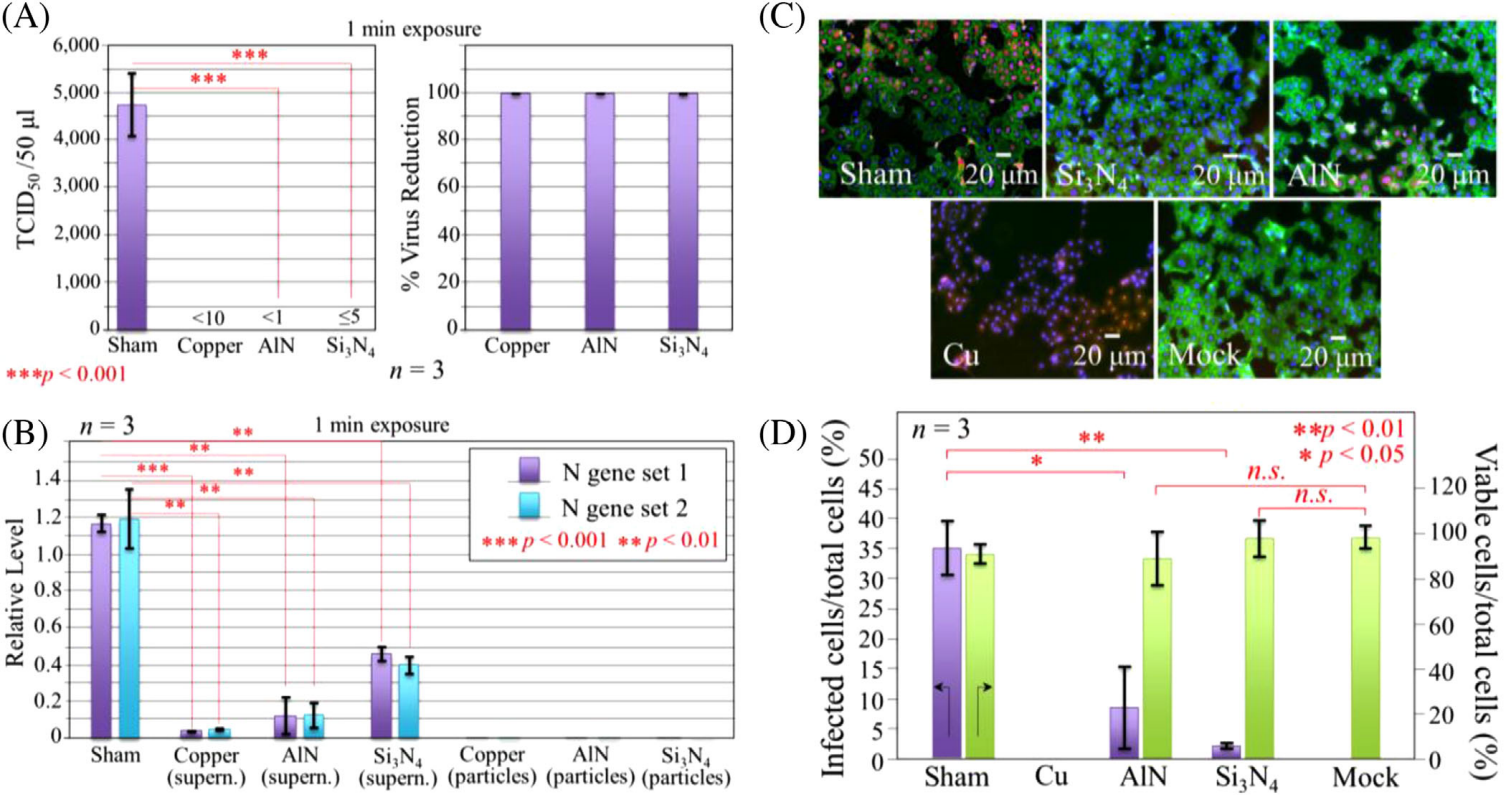
(A) TCID_50_/50 μL and % reduction plots by TCID_50_ assay (based on the Reed‐Muench method). (B) RT‐PCR tests to evaluate viral RNA using two sets of N gene primers; a comparison is given using evaluations of supernatants and powders with viral RNA from virions simply suspended in water. (C) Fluorescence micrographs inoculated VeroE6/TMPRSS2 cells after staining: red, green, and blue stains visualize viral protein, F‐actin, and cell nuclei, respectively. (D) Quantification of fluorescence microscopy data given as % infected cells on total cells, namely, the percent fraction of red‐stained cells with respect to the total number of blue‐stained nuclei, and the percent fraction of viable cells on total cells, namely, the percent fraction of green‐stained cells with respect to the total number of blue‐stained nuclei. Labels in inset specify statistics (unpaired two‐tailed Student's test with n = 3)

Figure 1C shows immunofluorescence imaging results on inoculated cells. The envelope antibody of the anti‐SARS coronavirus stained red; viable cell F‐actin (phalloidin‐stained) green; and cell nuclei (DAPI‐stained) blue. Micrographs showing fluorescence in Figure 1C compare the sham (negative) control VeroE6/TMPRSS2 cell population with populations inoculated with supernatant virions exposed to Si_3_N_4_, AlN, and Cu (see labels). The synthesis of viral protein, visualized by red‐fluorescent signals, imaged the sham sample cells extensively infected by the virus. As expected, as‐cultured VeroE6/TMPRSS2 cells unexposed to virions (mock sample) showed no red staining. A striking result was that cells inoculated with supernatant treated with Si_3_N_4_ and, to a lesser extent, with AlN, were viable and showed a low fraction of infected cells. On the other hand, cells infected with Cu‐treated viral supernatant were essentially dead (see complete lack of F‐actin), clearly indicating that it was free copper ions in the cells having toxic effects, and not viral infection, that caused cell death.^7^ We confirmed this using in situ Raman spectroscopy (see Supporting Information). In a quantitative plot of fluorescence microscopy results (Figure 1D), ∼35% fraction of cells in the sham sample (negative control) were infected. Comparatively, cells inoculated with Si_3_N_4_ supernatants showed only 2% infection and with AlN supernatants showed 8% infection (see Supporting Information for experimental procedures).

Our work revealed two pivotal aspects of Si_3_N_4_ surface chemistry that likely play fundamental roles in inactivating SARS‐CoV‐2: (a) protonation of the amino groups creates Si_3_N_4_ surface sites Si–NH_3_^+^ that resemble N‐terminals of lysine, C–NH_3_^+^, the cell side viral receptor; and, (b) hydrolytically eluted ammonia from the Si_3_N_4_ surface as a strong virucidal compound. Figure 2 (center) draws the interaction between virus and bioceramic surface in aqueous environment. At pH 7.4, positively charged viral envelope/membrane proteins are strongly attracted to the Si_3_N_4_ surface (see Supporting Information). The left panel depicts similarity between protonated amine and the lysine N‐terminal. As is the case with hepatitis B and influenza A,^5^, ^8^ an extremely effective “competitive binding” effect on SARS‐CoV‐2 occurs. Once in contact with the virus, eluted ammonia gas penetrates the virions and cuts through the RNA backbone^9^ (see Figure 2, right panel). The combination of RT‐PCR results and fluorescence microscopy suggest that SARS‐CoV‐2 inactivation takes place through a sequence of events: virions are first electrically trapped, locked by “competitive binding,” and then killed by “ammonia poisoning.” Such a scenario could be referred to as “catch and kill.”

**FIGURE 2:**
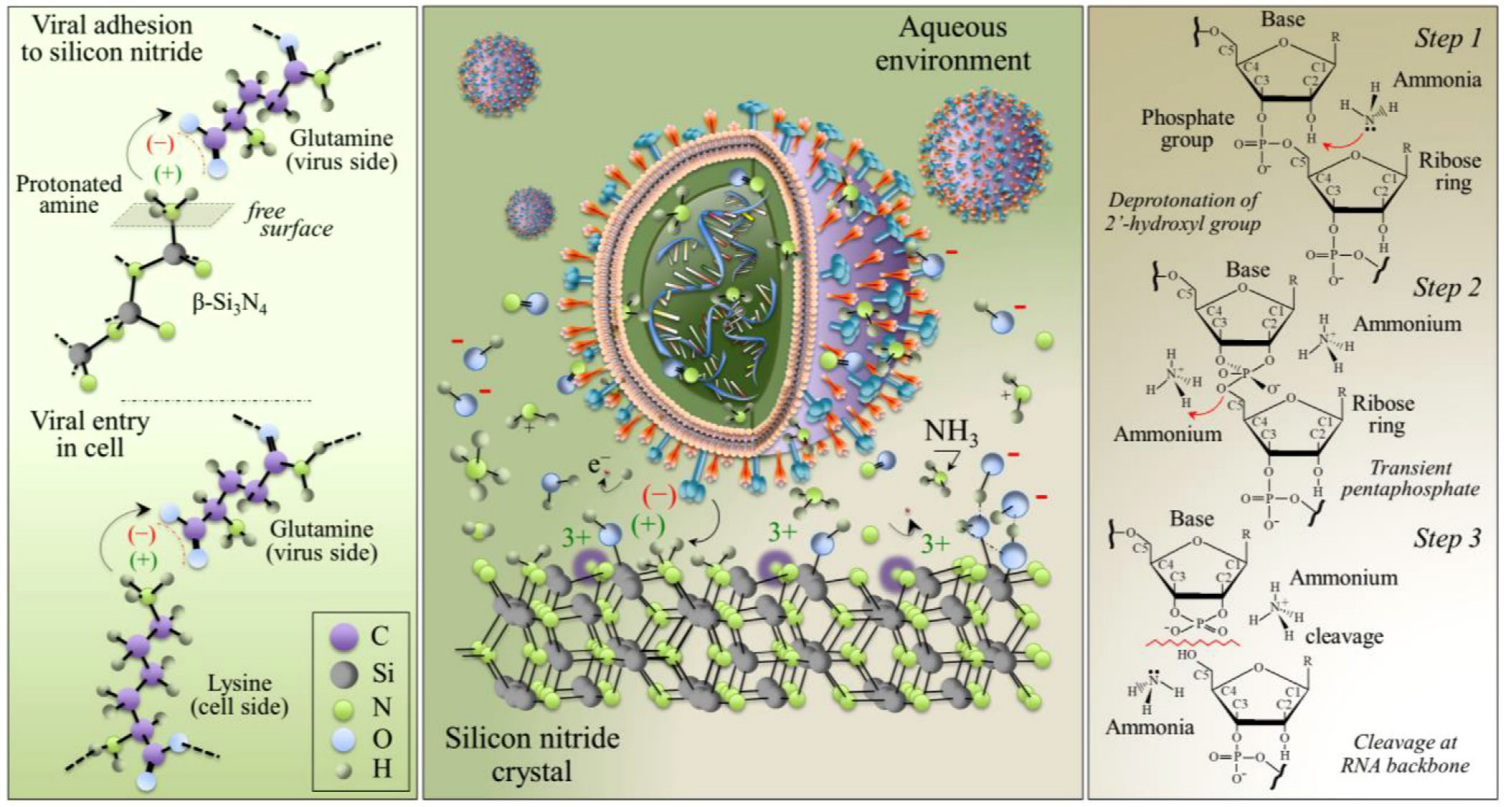
The “catch and kill” mechanism. *Central panel*: Draft of the electrochemical interaction between Si_3_N_4_ surface and SARS‐CoV‐2 virions (envelope and membrane proteins are electrostatically attracted at the negatively charged Si_3_N_4_ surface while protonated amines, which resemble cell lysine N‐terminal receptors, link with the spike protein and lock the virions; once the virion is “caught” and locked on the Si_3_N_4_ surface, eluted NH_3_ gas freely penetrates envelope proteins and “kills” it). *Left panel*: Draft of electrochemical “binding competitive” interactions between protonated amine groups on the surface of Si_3_N_4_ and lysine N‐terminals in cells. *Right panel*: RNA cleavage by ammonia species occurs in three successive steps including the deprotonation of backbone 2′‐hydroxyls, the formation of a transient pentaphosphate group, and the final RNA cleavage by alkaline transesterification

Results confirm SARS‐CoV‐2 inactivation was almost instantaneous upon contact with Cu, AlN, and Si_3_N_4_, but only the latter compound proved completely safe to host cells. The bioceramic, Si_3_N_4_, is thus a primary candidate to replace toxic and allergenic compounds in long‐term environmental sanitation.^10^ The use of micron‐sized Si_3_N_4_ particles in disinfectant sprays or their direct embedment in personal protective equipment fabrics (facemasks, surgical drapes, and other garments) in hospitals could limit viral transmission for both health workers and patients. As neither anion‐ nor cation‐side surface chemistry of Si_3_N_4_ will affect human health, even in the long term, this bioceramic has potential as an invaluable tool in fighting the SARS‐CoV‐2 pandemic.

## Supporting information

Supporting information
