# Supplementary material for: Instantaneous “catch‐and‐kill” inactivation of SARS‐CoV‐2 by nitride ceramics
Source: Clin Transl Med. 2020 Oct 18;10(6):e212. doi: 10.1002/ctm2.212 (PMC7568850; doi:10.1002/ctm2.212)
Supplement: Supplementary file 1 — Supporting information [file CTM2-10-e212-s001.docx]

Supplemental Information

Instantaneous “catch-and-kill” inactivation of SARS-CoV-2

by nitride ceramics

Giuseppe Pezzotti,^1,2^* Eriko Ohgitani,^2^ Masaharu Shin-Ya,^2^ Tetsuya Adachi,^3^ Elia Marin,^1,3^ Francesco Boschetto,^1,3^ Wenliang Zhu,^1^ and Osam Mazda,^2^*

# *Methods*

# Si_3_N_4_, Cu, and AlN powders were acquired from commercial sources (SINTX Technologies, Inc., Salt Lake City, UT USA, FUJIFILM Wako Pure Chemical Corporation, Osaka, Japan, and Tokuyama Co., Yamaguchi, Japan, respectively). The Si_3_N_4_ powder had an average particle size of 0.8 ± 1.0 μm. As-received Cu powder (USP grade 99.5% purity) granules were comminuted to achieve a particle size comparable to the Si_3_N_4_. AlN powder had an average particle size of 1.2 ± 0.6 μm as received, which was comparable to Si_3_N_4_.

VeroE6/TMPRSS2 cells (Japanese Collection of Research Biosources Cell Bank, National Institute of Biomedical Innovation, Osaka, Japan) were used in the viral assays. Cells were grown in Dulbecco’s modified Eagle’s minimum essential medium (DMEM) (Nissui Pharmaceutical Co. Ltd., Tokyo, Japan) supplemented with G418 disulfate (1 mg/ml), penicillin (100 units/mL), streptomycin (100 μg/mL), 5% fetal bovine serum, and maintained at 37°C in a 5% CO2 / 95% in a humidified atmosphere. The SARS-CoV-2 (Japan/AI/I- 004/2020; Japan National Institute of Infectious Diseases, Tokyo, Japan) viral stock was propagated using VeroE6/TMPRSS2 cells at 37°C for 2 days.

Fifteen weight percent (15 wt.%) of the Si_3_N_4_, Cu, and AlN powders were separately dispersed in 1 mL of PBS(-), followed by the addition of the viral suspension (2 x 10^5^ median tissue culture infectious dose (TCID_50_) in 20 μL). Due to the higher density of the Cu powder, its volumetric fraction was approximately one-third of the Si_3_N_4_. Mixing was gently performed at room temperature for 1 min by slow manual rotation or for 10 min using a rotation machine. After exposure, the powders were pelleted by centrifugation (2400 RPM 2 mins) followed by filtration through a 0.22 μm filter (Hawach Sterile PES Syringe Filter, HAWACH SCIENTIFIC CO., LTD., Xi’an, China). Supernatants were collected, aliquoted, and subjected to TCID_50_ assays and real-time RT-PCR.

Experiments were performed in triplicate including sham-treated virus suspension that was not exposed to any powder. A confluent monolayer of VeroE6/TMPRSS2 cells in a 96-well plate was inoculated with 50 μL/well of each virus suspension in a tenfold serial dilution with 0.5% FBS DMEM (*i.e*., maintenance medium). Viral adsorption at 37°C for 1 h was made with gently shaking every 10 min. Afterward, 50 μL/well of the maintenance medium was added. The plate was incubated at 37°C in a 5% CO2 /95% humidified atmosphere for 4 days. The cytopathic effect (CPE) of the infected cells was observed under a phase-contrast microscope. The cells were subsequently fixed by adding 10 μL/well of glutaraldehyde followed by staining with 0.5% crystal violet. The TCID_50_ was calculated according to the Reed-Muench method.

After exposure to the powders, 140 μL of the supernatants were used for viral RNA extraction. RNA was also extracted from the surfaces of the centrifuged and filtered powders. RNA purification was performed using a QIAamp Viral RNA Mini kit (QIAGEN, Germantown, MD, USA). An aliquot of 16 μL of isolated RNA was reverse-transcribed using ReverTra Ace^®^ qPCR RT Master Mix (Toyobo, Shiga, Japan). Quantitative real-time PCR was performed using a Step-One Plus Real-Time PCR system (Applied Biosystems, Foster City, CA, USA) and two sets of primers/probes specific for viral N gene. The amplification protocol consisted of 50 cycles of denaturation at 95°C for 3 s and annealing and extension at 60°C for 20 s.

Vero E6/TMPRSS2 cells on cover glass were inoculated with 200 μL of virus supernatant. After viral adsorption at 37°C for 1 h, the cells were incubated with the maintenance medium in a CO_2_ incubator for 7 h. For the detection of infected cells, they were washed with TBS (20 mM Tris-HCl pH 7.5, 150 mM NaCl) and fixed with 4% PFA for 10 min at room temperature (RT) followed by membrane permeabilization with 0.1% Triton X in TBS for 5 min at RT. The cells were blocked with 2% skim milk in TBS for 60 min at RT and stained with anti-SARS Coronavirus envelope (Rabbit) antibody (Dilution =1:100) (ProSci Inc., Poway, CA, USA) for 60 min at RT. After washing with a buffer, cells were incubated with an Alexa 594 Goat Anti- Rabbit IgG (H+L) (1:500) (Thermo Fisher Scientific, Waltham, MA, USA) and Alexa 488 Phalloidin (1:50) (Thermo Fisher Scientific, Waltham, MA, USA) for 60 min at RT in the dark. ProLongTM Diamond Antifade Mountant with DAPI (Thermo Fisher Scientific) was used as a mounting medium. The staining was observed under a fluorescent microscope BZX710 (Keyence, Osaka, Japan). The total cells, Phalloidin-staining cells, and protein-expressing cells were counted using the Keyence BZ-X Analyzer.

Vero E6/TMPRSS2 cells were infected with 200 μL of each virus suspension on glass sites. After viral adsorption at 37°C for 1 h, the infected cells were incubated with the maintenance medium in a CO_2_ incubator for 4 h and fixed with 4% paraformaldehyde for 10 min at RT. After washing with distilled water twice, infected cells were air-dried and *in situ* analyzed using a highly sensitive Raman spectroscope (LabRAM HR800, Horiba/Jobin-Yvon, Kyoto, Japan) with a 20× optical lens. It operated in microscopic measurement mode with confocal imaging in two dimensions. A 532 nm excitation source operating at 10 mW was used.

The Student's *t*-test determined statistical significance for *n*=3 and at a *p*-value of 0.01 using Prism software (GraphPad, San Diego, CA USA).

*TCID_50_ assay and RT-PCR test results after 10 min exposure*

**
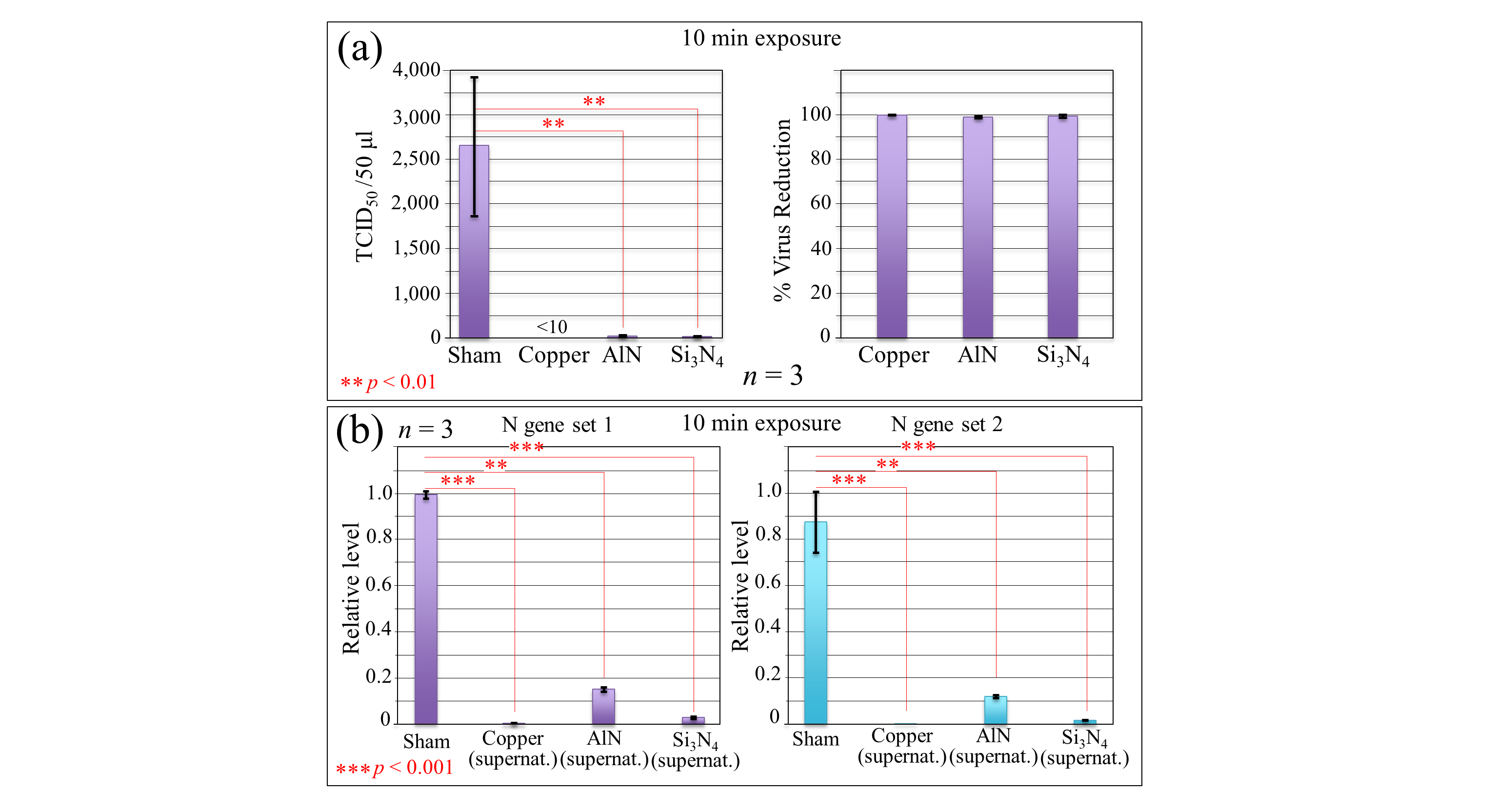
**

**Figure S-1:** Inactivation of SARS-CoV-2 by nitride powders; virus suspensions were treated with 15 wt.% Cu, AlN, and Si_3_N_4_ powders in an aqueous medium at room temperature for 10-min. The control virus was treated identically in water without the addition of any powder (sham). After light centrifugation and filtration, supernatants were subjected to: (a) TCID_50_ assay and (b) RT-PCR test. Regarding (a), the Reed-Muench method was used to determine the virus titers. Regarding (b), results are given for viral N gene “set 1” and “set 2” primers. In both type of tests, statistics are given in the inset according to unpaired two-tailed Student’s *t*-test (*n*=3).

*Surface charge, the isoelectric point (IEP) of of Si_3_N_4_, and its viral catching efficiency*

Zeta potential measurements were performed using an electrokinetic analyzer (SurPASS, Anton-Paar USA, Ashland, VA). A background electrolyte of 1 mM HCl, which exhibited a natural pH of 5.5, was used in all experiments. Experiments were divided into two runs. The first run took measurements across a pH range of 5.5–3 using auto-titration of 0.1 M HCl solution to control pH. The second run used a new solution of background electrolyte for measurements across a pH range of 5.5-10 and auto-titration of 0.1 M NaOH solution to control pH. Each run contained two material samples. Observed streaming potentials were converted into zeta potentials using the Helmholz-Smoluchowski equation. Figure S-2 shows the results of IEP measurements on the present Si_3_N_4_ material. The IEP was in the range 4.5 ± 0.1. A comparison between the zeta potential curve of the Si_3_N_4_ powder and the recently reported IEP values for SARS-CoV-2 proteins is given in Fig. S-2 [S1]. An examination of the charges of different viral proteins shows that P0DTC2, namely the viral spike glycoprotein playing the key-role in docking interaction with ACE2 receptor of human cells, bears negative charge at homeostatic pH=7.4. Its IEP is reported to be 5.35 [S1]. In the virion structure, these spikes locate at the outermost layer and are expected to interact with the amine sites on the surface of Si_3_N_4_, as explained in the main text. Conversely, the both envelope and membrane proteins charge positive and possess significantly higher IEPs, comprised in the ranges 6.2~7.2 and 9.3~9.6, respectively [S1]. In summary, the graph in Fig. S-2 shows that, at homeostatic pH=7.4, the surface of the present Si_3_N_4_ strongly attracts the proteins of both envelop and membrane of the SARS-CoV-2 virions. Once the virions are fatally “caught” by electrical charge attraction, the P0DTC2 spikes find the Si_3_N_4_ surface sites, Si–NH_3_^+^, that resemble the N-terminals of lysine, C–NH_3_^+^ (i.e., the cell-side viral receptors), which lock the virions in “competitive binding”.

**
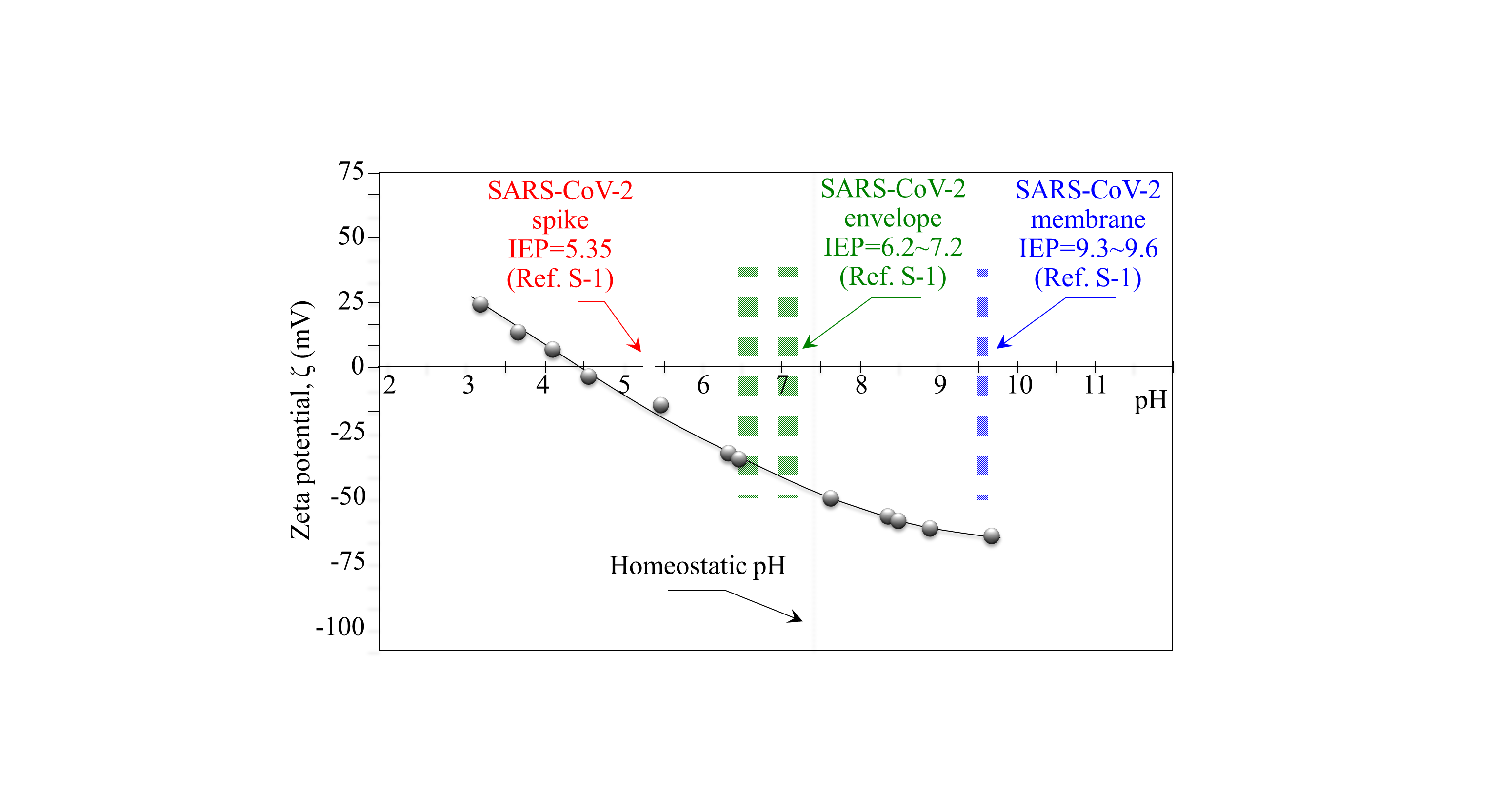
**

**Figure S-2:** Results of IEP measurements on the present Si_3_N_4_ powder in comparison with the IEPs of proteins in the membrane, envelope, and spike of the SARS-CoV-2 virions, according to Ref. [S1].

*In situ Raman spectroscopic assessments of virally inoculated cells*

Raman spectroscopy examined *in situ* VeroE6/TMPRSS2 cells exposed to different supernatants to assess biochemical cellular changes due to infection and ionic (*i.e.*, Cu and Al) toxicity. Figure S-3 shows Raman spectra in the frequency range 700~900 cm^-1^ for: (a) uninfected VeroE6/TMPRSS2 cells (mock), and cells inoculated with supernatants containing virions exposed for 10 mins to (b) Si_3_N_4_, (c) AlN, (d) Cu (positive control), and (e) no antiviral compounds (sham negative control). Of fundamental importance are the vibrational bands of ring breathing and H-scissoring of the indole ring of tryptophan (at 756 and 875 cm^-1^ [S2], labeled as T_1_ and T_2_, respectively). Tryptophan plays a vital role in protein synthesis and the generation of molecules for various immunological functions. Its stereoisomers serve to anchor proteins within the cell membrane [S3] and its catabolites possess immunosuppressive functions [S4]. The catabolism of tryptophan is triggered by a viral infection. This occurs via the enzymatic activity of indoleamine-2,3-dioxygenase (IDO) which protects the host cells from an over-reactive immune response. IDO reduces tryptophan to kynurenine and then to N’-formyl-kynurenine. An increase in IDO activity depletes tryptophan [S5]. Consequently, the intensity of the tryptophan bands (T_1_ and T_2_) is an indicator of these biochemical changes. Except for the Cu-treated sample, data in Fig. S-3(f) show an exponential decline in the combined tryptophan bands that correlates with the fraction of infected cells. (The chemical structure of N’-formyl-kynurenine is given in the inset for clarity.) The anomaly for copper provides further evidence of its toxicity. The VeroE6/TMPRSS2 cells consumed tryptophan to reduce Cu^2+^ and stabilize it as Cu^+^ [S6].

The Raman signals due to ring-stretching vibrations of adenine, cytosine, guanine, and thymine were found at 725, 795, 680, and 748 cm^-1^, and are labeled as A, Cy_1_, G, and Th, respectively, in Fig. S-3(a)~(e)) [S7]. These bands were preserved after virus exposure. However, there was an anomaly for lines representative of tyrosine at 642 and 832 cm^-1^ labeled as Ty_1_ and Ty_2_, respectively [S8] for cells infected with Cu-exposed virions. The ring-breathing band Ty_2_ of tyrosine was very weak compared to the other samples (*cf*. Fig. S-3(d) with (b)). Conversely, the C-C bond-related Ty1 signal remained strong. This suggests that the aromatic ring of tyrosine chelated the Cu ions [S9]. This explains why only the tyrosine ring-breathing mode was reduced while the C-C signal remained unaltered. Three possible Cu(II) chelating conformations in tyrosine are given in Fig. S-3(g) [S9,S10].

For VeroE6/TMPRSS2 cells exposed to virions treated with AlN (Fig. S-3(c)), the tryptophan T_1_ and T_2_ bands were preserved, but the bands at 615 and ~700 cm^-1^ due to ring bending in DNA cytosine (labeled as Cy_2_ and Cy_3_, respectively, in Fig. S-3) almost vanished [S7]. Their disappearance is due to either progressive internucleosomal DNA cleavage or from the formation of complexes, and both are related to toxicity [S11, S12]. The loss of the cytosine signals is interpreted as a toxic effect by Al ions, although it is far less critical than copper. Al^3+^interacts with carbonyl O and/or N ring donors in nucleotide bases [S13, S14] and selectively binds to the backbone of the PO_2_ group and/or to the guanine N-7 site of the G-C base pairs by chelation [S15, S16].

Unlike exposure of the VeroE6/TMPRSS2 cells to Cu and AlN supernatants, which resulted in moderate to severe toxicity, Si_3_N_4_ invoked no modifications of tryptophan, tyrosine, and cytosine. The morphology of the spectrum for the Si_3_N_4_ viral supernatant closely matched that of the uninfected mock suspension (*cf*. Figs. S-3(a) and (b)).

The persistence of human coronaviruses on common materials (*e.g.*, metal, plastic, paper, and fabric) and touch surfaces (*e.g.*, knobs, handles, railings, tables, and desktops) can contribute to the nosocomial and social spread of disease [S17, S18]. Warnes *et al.* reported that at room temperature with 30%-40% humidity, the pathogenic human coronavirus 229E (HuCoV-229E) remained infectious in a lung cell model after at least 5 days of persistent viability on a variety of materials, such as Teflon, polyvinyl chloride, ceramic tile, glass, stainless steel, and silicone rubber [S19]. These investigators also showed rapid HuCoV-229E inactivation (within a few minutes) for simulated fingertip contamination on Cu surfaces. Cu ion release and the generation of reactive oxygen species (ROS) were involved in viral inactivation; and increased contact time with copper and brass surfaces led to greater non-specific fragmentation of viral RNA, indicating irreversible viral inactivation [S19]. More recently, Doremalen *et al*. showed surface stability of both SARS-CoV-1 and SARS-CoV-2 virus on plastic, cardboard, stainless steel, and even Cu surfaces for 4~72 hours after application [S20]. While breathable N95-rated masks can capture particulates before they can be inhaled, SARS-CoV-2 virus particles remain active in mask filters for up to 7 days [S21]. Contact killing of viruses, such as observed on Cu surfaces is, therefore, receiving renewed interest as a disease mitigation strategy [S22].

The present work is the first to show that compounds capable of endogenous nitrogen-release, such as Si_3_N_4_ and AlN, can inactivate the SARS-CoV-2 virus at least as effectively as Cu. These results suggest that multiple antiviral mechanisms may be operative, such as RNA fragmentation, and in the case of Cu, direct metal ion toxicity; but while Cu and AlN supernatants demonstrated strong and partial cellular lysis, respectively, Si_3_N_4_ provoked no metabolic alterations. The Raman spectrum of VeroE6/TMPRSS2 cells exposed to the Si_3_N_4_ viral supernatant was like that of the uninfected sham. These findings indicate that while Si_3_N_4_, Cu, and AlN were all capable of inactivating the SARS-CoV-2 virus, Si_3_N_4_ was the safest compound for the tested cell model.

**
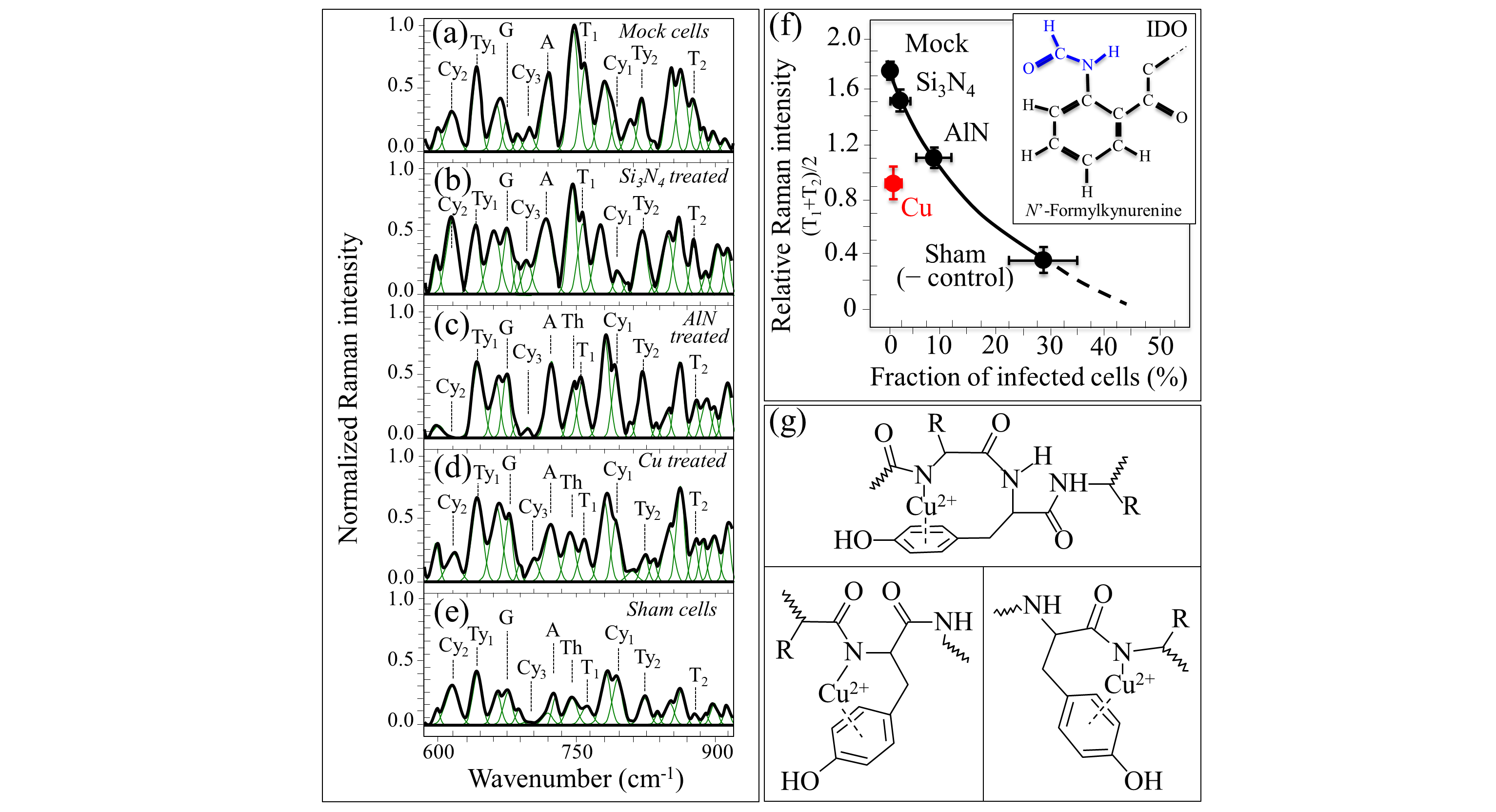
**

**Figure S-3:** Raman spectra of: (a) uninfected cells (mock sample unexposed to virions), and cells infected with SARS-CoV-2 virions exposed for 10 min to (b) Si_3_N_4_, (c) AlN, and (d) Cu; in (e), Raman spectrum of cells infected by virions merely suspended in water (sham negative control sample). In (f), a plot of the average intensity of the two tryptophan T_1_ and T_2_ bands (at 756 and 875 cm^-1^, respectively) as a function of the fraction of infected cells; labels locate pristine cells (mock sample), cell sample infected by virions exposed to only water (sham sample or negative control), and cell samples exposed for 10 min to water with different particles in suspension (*cf*. labels); in inset, the structure of N’-formylkynurenine, an intermediate in the catabolism of tryptophan upon enzymatic IDO reaction. In (g), three possible conformations of tyrosine-based peptides that can justify the disappearance of ring vibrations in tyrosine (Ty_2_ band) upon chelation of Cu(II) ions.

**Acknowledgements**

The authors gratefully thank the National Institute of Infectious Diseases for providing the SARS-CoV-2 virions. The Raman experiments were partly carried out at the Kyoto Municipal Institute of Industrial Technology and Culture. The authors gratefully thank Professors Narisato Kanamura and Toshiro Yamamoto for their important discussions.

**References:**

[S1] K. Mallik, *Preprints* (www.preprints.org) doi:10.20944/preprints202005.0270.v1

[S2] G. Zhu et al., Spectrochim. Acta - Part A Mol. Biomol. Spectrosc. 78 (2011) 1187-1195.

[S3] A.J. De Jesus, T.W. Allen, Biochim. Biophys. Acta - Biomembr. 1828 (2013) 864-876.

[S4] A.L. Mellor, D.H. Munn, J. Immunol. 170 (2003) 5809-5813.

[S5] J.M. Fox et al., J. Gen. Virol. 94 (2013) 1451-1461.

[S6] M. Okada, T. Miura, J. Inorg. Biochem. 159 (2016) 45-49.

[S7] C. Otto et al., J. Raman Spectrosc. 17 (1986) 289-298.

[S8] H. Li, C.J. Wurrey, G.J. Thomas, J. Am. Chem. Soc. 114 (1992) 7463-7469.

[S9] H. Peifeng, C. Sorensen, M.L. Gross, J. Am. Soc. Mass Spectrom. 6 (1995) 1079-1085.

[S10] M. Vandenbossche et al., Green Mater. 3 (2015) 1-9.

[S11] E. Brauchle et al., Sci Rep. 4 (2014) 4698.

[S12] I. Notingher et al., J. R. Soc. Interface 1 (2004) 79–90.

[S13] W.R. Harris et al., J. Toxicol. Environ. Health 48 (1996) 543–568.

[S14] V.G. Kanellis, C.G. dos Remedios CG, Biophys. Rev. 10 (2018) 1401–1414.

[S15] K.S.J. Rao et al., BBA - Gene Struct. Expr. 1172 (1993) 17–20.

[S16] R. Ahmad et al., J. Biomol. Struct. Dyn. 13 (1996) 795–802.

[S17] J.A. Otter et al., Am. J. Infect. Control 41 (2013) S6.

[S18] J.A. Otter, S. Yezli, G.L. French, In: G. Borkow, editor. Use Biocidal Surfaces for Reduction of Healthcare Acquired Infections. Heidelberg New York Dordrecht London: Springer Cham; 2014. p. 1–215.

[S19] S.L. Warnes, Z.R. Little, C.W. Keevil, MBio 6 (2015) 1–10.

[S20] N. van Doremalen, T. Bushmaker, D.H. Morris, N. Engl. J. Med. (2020) Correspond:1–3.

[S21] A. Chin et al., 5247 (2020) 2020.03.15.20036673.

[S22] G. Grass, C. Rensing, M. Solioz, Appl. Environ. Microbiol. 77 (2011) 1541–1547.
